# Efficacy and safety of modified Yupingfeng formula in treating allergic rhinitis: A protocol for systematic review and meta analysis

**DOI:** 10.1097/MD.0000000000023698

**Published:** 2020-12-18

**Authors:** Chao Liao, Ting Liu, Zhen Zeng, Dandan Wang, Guangjun Tang, Huan Wang, Li Tian

**Affiliations:** Hospital of Chengdu University of Traditional Chinese Medicine, Sichuan Province, PR China.

**Keywords:** Allergic rhinitis, modified Yupingfeng formula, protocol, systematic review and meta-analysis

## Abstract

**Background:**

Allergic rhinitis (AR) is a non-infectious chronic nasal mucosal disease mediated mainly by IgE, which affects 40% of the global population and has a recurrence rate of more than 50%. The modified Yupingfeng formula (MYPFF) is widely used in the treatment of allergic rhinitis in China. However, there is no evidence-based medical evidence for the efficacy and safety of MYPFF in the treatment of allergic rhinitis.

**Methods:**

Database as China National Knowledge Infrastructure, Chinese Biomedical Literature Database, Chinese Scientific Journal Database (VIP database), Wan-Fang Database, the Cochrane Central Register of Controlled Trials (CENTRAL), PubMed, EMBASE, and Web of Science will be searched for relevant literature from inception to September 2020. Data extraction will be performed on the obtained literature. Then RevMan V.5.3 will be used for the assessment of the risk of bias and data synthesis.

**Results:**

The results will be published in a peer-reviewed journal.

**Conclusion:**

The conclusion of the study will provide an evidence to efficacy and safety of MYPFF in treating allergic rhinitis, which will be of significant meaning for further research and clinical practice.

**OSF registration number:**

10.17605/OSF.IO/RV9P4.

## 1 Introduction

Allergic rhinitis (AR) is a non-infectious chronic nasal mucosal disease mediated mainly by IgE, whose main clinical manifestations are nasal itching, sneezing, watery nasal discharge, and/or nasal congestion.^[1]^ It affects 40% of the global population and has a recurrence rate of more than 50%.^[1]^ It is a global health problem that causes major diseases and disabilities worldwide.^[2]^ In the past 6 years, the prevalence rate of AR in China has increased from 11.1% to 17.6%,^[2]^ and the medical resource consumption is about 14.79 billion US dollars,^[3]^ causing great social and economic pressure. Currently, the main drugs for the treatment of allergic rhinitis are H1 antihistamines, nasal steroid hormones, and leukotriene receptor antagonists. The control and safety of nasal symptoms have been verified, but there are still deficiencies in the control of the recurrence of allergic rhinitis.

Traditional Chinese medicine has a multi-target and multi-link comprehensive regulatory effect.^[4–6]^ Modern pharmacological studies^[7]^ have shown that this prescription can improve allergic symptoms of allergic rhinitis by inhibiting mast cell activation. Astragalus in Yupingfeng can not only improve the bodys immunity when the immune system is low, but also play an immunosuppressive role in reducing the production of pro-inflammatory factors when the inflammation is persistent, thus playing a bidirectional regulatory role.^[8]^ Fangfeng has anti-inflammatory, antioxidant, and immunomodulatory effects.^[9]^ Atractylodes atractylodes has the effect of repairing mucosa and anti-inflammatory.^[10]^ A study on the treatment of allergic rhinitis based on network pharmacology found that HIF-1 signaling pathway, IL-17 signaling pathway, VEGF signaling pathway, Ca2+ signaling pathway, and ErbB signaling pathway are important pathways in the pathogenesis of allergic rhinitis.^[11]^ With literature reported that oxygen plays an important role in the pathogenesis of allergic rhinitis, it can make the IL - 17 a levels in nasal mucosa, and IL - 17 a mainly produced by the differentiation of the auxiliary Th17, Th17 cells in a variety of inflammatory and autoimmune disease plays an important role, the secretion of IL - 17 in hypoxia environment by mediating cell within the nf-kappa B and P13K/AKT pathway, activate the downstream of HIF - 1 alpha and VEGF signaling molecules. Activated HIF-1 is directly involved in angiogenesis and aggravates allergic airway inflammation by regulating the expression of other growth factors.^[12]^ High expression of HIF-1 promotes the expression of VEGF, which is a potent stimulant of inflammatory responses, airway remodeling, and physiological disorders.^[13]^

The purpose of this study is to provide evidence-based medical evidence for the efficacy and safety of modified Yupingfeng formula in the treatment of allergic rhinitis by systematic evaluation and meta-analysis.

## 2 Methods

### 2.1 Study registration

This meta-analysis was registered on OSF (registration number: DOI 10.17605/OSF.IO/RV9P4) and will be carried out under the guidance of Preferred Reporting Items for Systematic

Review and Meta-Analysis Protocols (PRISMA-P)^[14]^ and the Cochrane Handbook.^[15]^

### 2.2 Types of studies

All randomized controlled trials (RCTs) about AR treated with MYPFF will be included in this systematic review. Semi-randomized control studies will be included when we are unable to find at least 5 eligible RCTs for the systematic review.

### 2.3 Type of participant

Patients diagnosed AR with regardless of sex, age, race, or educational and economic status, will be included in the review.

### 2.4 Type of interventions

Interventions included the use of modified Yupingfeng formula alone and combination with modern drugs. The control group received conventional modern medical treatment with drugs including antihistamines, glucocorticoids, antileukotrienes, chromones, intranasal decongestants, or combination drugs.

### 2.5 Type of outcome measures

The primary outcome includes effective rate, Total Nasal Symptom Score (TNSS), Visual Analog Scale (VAS), and Rhinoconjunctivitis Quality of Life Questionnaire (RQLQ). The secondary outcome includes adverse events, serum level change and recurrence rate.

### 2.6 Search strategy

Database as China National Knowledge Infrastructure, Chinese Biomedical Literature Database, Chinese Scientific Journal Database (VIP database), Wan-Fang Database, the Cochrane Central Register of Controlled Trials (CENTRAL), PubMed, EMBASE, and Web of Science will be searched for relevant literature from inception to September 2020. The search formula is as follows: (“Allergic rhinitis” OR “AR” OR “Bi Qiu”) AND (“ Yupingfeng ”OR“ Modified Yupingfeng ” OR “Yupingfeng formula” OR “Yupingfeng powder”) AND (“randomized clinical trial” OR “randomized controlled trial” OR “clinical trial”). The Chinese database will be retrieved by the corresponding retrieval method. Manually search some relevant journals, track relevant literature in clinical trial report papers or review references, and contact relevant researchers for relevant literature as far as possible.

### 2.7 Study screening and data extraction

**Figure 1:**
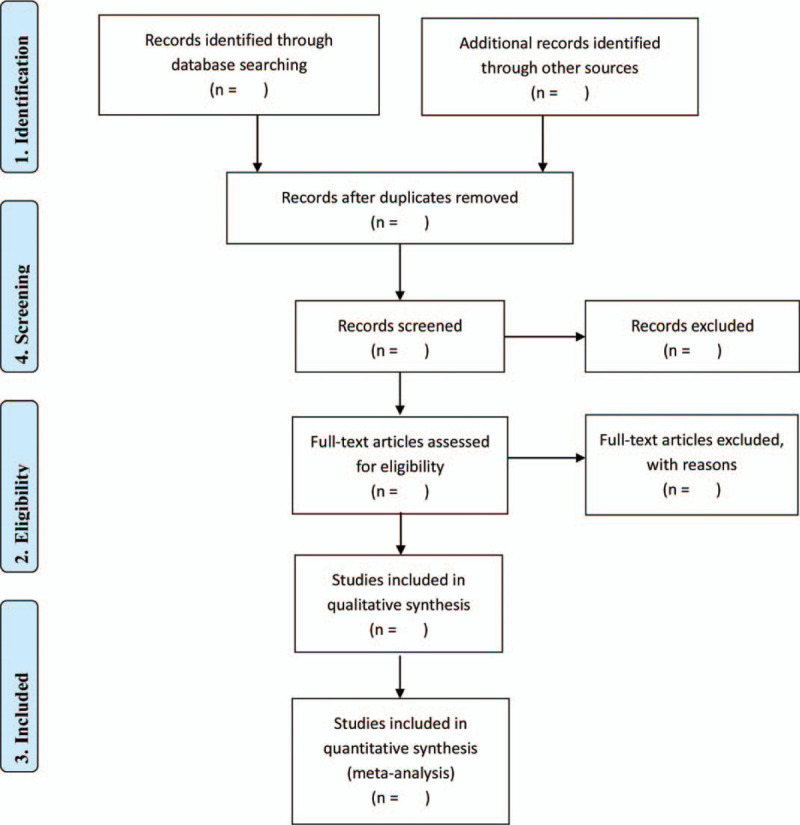
Flow chart of study selection.

The selection process will be carried out as flow chart follows (Fig. 1). Two independent authors will screen the titles and abstracts of all searched studies. Any disagreements will be solved through discussion with a third reviewer. Data from each article will be record on a designed data extraction sheet, which includes publication time, interventions for each group, sample size, randomization, blinding, outcome measures, adverse events, recurrent rate, and follow-up time.

### 2.8 Quality assessment of the studies

The quality of the articles included in the study will be evaluated according to the Cochrane Collaborations tool for assessing the risk of bias,^[12]^ which considers random sequence generation, allocation concealment, blinding of participants and personnel, blinding of outcome assessors, incomplete outcome data, selective reporting, and other sources of bias.

### 2.9 Data analysis

Rev-man 5.3 software provided by the Cochrane Collaboration will be used for statistical analysis. Odds ratio (OR) was used for enumeration data and standardized mean difference (SMD) was used for measurement data, both represented by 95% confidence interval (CI). The *x*^2^ test and the *I*^2^ statistic will be used for assessing heterogeneity. If there is no heterogeneity, the *I*^2^ value less than 50%, fixed-effect model would be used for statistical analysis. Otherwise, subgroup analysis would be carried out according to the source of heterogeneity, and statistical analysis was carried out on the random effect model with unknown source of heterogeneity.

### 2.10 Ethics and dissemination

No individual patient will be involved in this study, so it does not require ethical approval. The results from this review will be disseminated through peer-reviewed journals and conference reports.

## 3 Discussion

In China, the MYPFF is widely used in the treatment of allergic rhinitis. The 2018 Chinese Guidelines for the Diagnosis and Treatment of allergic rhinitis include TCM treatment as a guideline.^[16]^ It is pointed out that specific syndromes and drug recommendations have not been made due to the lack of large sample and multi-center randomized controlled studies.^[16]^ The purpose of this protocol is to provide evidence-based medical evidence for the efficacy and safety of MYPFF in the treatment of allergic rhinitis through systematic review and meta-analysis.

## Author contributions

Chao Liao, Ting Liu and Li Tian contributed to the design of the protocol. Ting Liu and Zhen Zeng developed the search strategy and were responsible for the search. Chao Liao and Huan Wang screened the included literature. Chao Liao and Dandan Wang imported the data into Revman. The data analysis was carried out by Dandan Wang and Guangjun Tang. The final draft was written by Chao Liao and Ting Liu. Li Tian is responsible for guiding the whole program and solving disagreements. All authors have read and approved the final version of the manuscript.

**Data curation:** Dandan Wang, Guangjun Tang.

**Formal analysis:** Dandan Wang, Guangjun Tang.

**Investigation:** Zhen Zeng, Dandan Wang, Guangjun Tang, Huan Wang.

**Methodology:** Chao Liao, Ting Liu, Guangjun Tang, Li Tian.

**Project administration:** Chao Liao, Li Tian.

**Software:** Zhen Zeng, Dandan Wang.

**Supervision:** Li Tian.

**Writing – original draft:** Chao Liao.

**Writing – review & editing:** Chao Liao, Ting Liu.
